# A Case of A Sleepy Taxi Driver Presenting with Narcolepsy

**DOI:** 10.7759/cureus.4901

**Published:** 2019-06-14

**Authors:** Jeffrey A Miskoff, Moiuz Chaudhri

**Affiliations:** 1 Internal Medicine, Jersey Shore University Medical Center, Neptune City, USA

**Keywords:** case report, motor vehicle, narcolepsy, off label, sodium oxybate, hypocretin, sleep disorder, excessive sleepiness

## Abstract

Narcolepsy is a sleep disorder, which can manifest in childhood, or adolescence by causing excessive daytime sleepiness, hallucinations, sleep attacks or cataplexy. Although presentation can vary, nearly all patients present with excessive daytime sleepiness (EDS). There is often a significant delay in diagnosis, which may lead to a misdiagnosis. Timely diagnosis and management may dramatically improve quality of life and symptoms. If missed, a patient may be at risk of accidents at home, work or while operating motor vehicle or machinery. This particular case describes a 42-year-old male, taxicab driver, who has been living with this undiagnosed condition his entire life along with examining real life consequences of his condition due to his occupation.

## Introduction

Narcolepsy is a rare sleep disorder which impairs regulation of the sleep-wake cycle. Most patients present with excessive daytime sleepiness (EDS) occurring in isolation or in combination with features of abnormal rapid eye movement (REM) such as cataplexy, hypnagogic hallucinations, and sleep paralysis [1]. One of the biggest challenges associated with narcolepsy stems from lack of clinical experience. Research suggests that there is up to a 10-year delay in diagnosing narcolepsy which may impact patients and their families adversely [1]. Also, multiple clinicians may misdiagnose or miss the diagnosis all together for narcolepsy. Therefore, objective of this case is to highlight the importance of continued education to reduce the risk of delay in diagnosis along with reviewing current therapies and their efficacy.

## Case presentation

A 42-year-old male presented to our care in April 2018 for evaluation of excessive daytime sleepiness experiencing since childhood. In addition to EDS, the patient described falling asleep at a moment's notice especially after entering a state of extreme relaxation. According to the patient, he has experienced sleepiness while driving typically when stopped at a traffic light. Over the years, sleepiness while driving led to multiple motor vehicle accidents and latest accident occurred in early April 2018 requiring a visit to the emergency room. During this visit, his history of multiple motor vehicle accidents due to excessive sleepiness were documented resulting in his driving privileges being suspended until an appropriate evaluation with a sleep specialist. In addition to feeling sleepy, he reported experiencing probable episodes of cataplexy triggered by laughter mostly at comedy clubs. In light of these complaints, the patient was sent for a further workup including the STOP-BANG test (snoring, tiredness, observed apnea, blood pressure, body mass index, age, neck circumference and gender) and the Epworth Sleepiness Scale (ESS). In addition, a nocturnal polysomnogram (NPSG) and a multiple sleep latency test (MSLT) were also completed.

The STOP-Bang questionnaire is the best validated tool for screening obstructive sleep apnea (OSA) patients for preoperative risk and includes eight questions which are answered with a Yes or No. Scores of 3 to 4 are classified as an intermediate risk whereas score of ≥5 represents high risk of postoperative complications including extended hospital stay. Clinical evidence suggests that a score of ≥3 out of 8 is highly sensitive for diagnosing OSA [2]. Our patient scored 6 out of 8.

The ESS is a subjective test utilized to measure patient’s sleepiness. It includes eight scenarios in which the patient rates their tendency to become sleepy on a scale of 0 (no chance of dozing), 1 (slight chance of dozing), 2 (moderate chance of dozing), and 3 (high chance of dozing) [3]. These eight scenarios are sitting and reading, watching television, sitting inactive in a public place, as a passenger in a car for an hour without a break, in a car while stopped for a few minutes in traffic, lying down to rest in the afternoon, sitting and talking to someone, and sitting quietly after a lunch without alcohol. Our patient scored 22 which is highly associated with pathologic sleepiness because a score of greater than 15 suggests that the patient is excessively sleepy and should be treated [4,5].

Nocturnal polysomnogram (NPSG) is a sleep study which records brain waves, oxygen levels, heart rate and breathing patterns along with eye and extremity movement as the patient sleeps [6,7]. According to the results of NPSG, the patient slept 380.5 minutes out of 435.0 minutes in bed for a sleep efficiency of 87.47%. His sleep latency was recorded at 21.0 minutes with persistent sleep of 21.0 minutes. In addition, REM sleep and latency were logged at 16% and 98 minutes, respectively. Furthermore, the sleep study illustrated that the patient experienced 33 obstructive apneas, zero central and mixed apneas, 201 hypopneas, and apnea-hypopnea index of 36.9 events/hour; consistent with severe OSA. It is important to note that the patient's body mass index (BMI) was recorded as 40 (kg/m^2^) which is associated with morbid obesity.

An MSLT is the primary diagnostic tool for narcolepsy and is typically performed following an NPSG to measure sleep latency [1]. Sleep latency can be described as amount of time it takes to go from wakefulness to entering sleep. In addition, it also measures sleep onset REM periods (SOREMPs) which illustrates how quickly the patient enters REM sleep. This test typically includes four or five naps lasting for 20 minutes each. Results of the MSLT consisted of four nap trials with a sleep latency of 0, 3, 0 and 6.5 minutes, respectively and a mean sleep latency of 2.4 minutes. Furthermore, results indicated one SOREMPs by our patient which was achieved in the second nap. The diagnostic criterion requires an MSL of ≤8 minutes and ≥2 SOREMPs on an MSLT for an objective diagnosis of narcolepsy [1].

Based on the workup, the patient was diagnosed with narcolepsy which has been successfully managed with 4.5 g of sodium oxybate taken at bedtime and repeated 3 to 4 hours later. Furthermore, addition of 10 mg dextroamphetamine taken twice a day has worked well for this gentleman. In addition to narcolepsy, the patient was treated for obstructive sleep apnea with continuous positive airway pressure (CPAP) which has provided tremendous additional clinical benefit to the patient allowing him to obtain more refreshing sleep. On a follow-up visit, data from the patient’s CPAP download, set at 5-15 cm of water (cmH2O), proved patient’s compliance of 93.3% of the nights with more than 4 hours of usage. Moreover, the patient’s Apnea Hypopnea Index (AHI) dropped to 2.2 events per hour sleep, which is within the normal limits [8].

## Discussion

Narcolepsy is one of the most common causes of excessive daytime sleepiness whose etiology is multifactorial [9]. Although clinical evidence strongly points towards loss of the neuropeptide, hypocretin, as being the main culprit, the role of genetics and other factors should not be minimized [10]. Original function of hypocretin is associated with increasing activity, inhibiting REM sleep and promoting wakefulness. One of the most common complaints is excessive daytime sleepiness, not being able to sleep at night along with experiencing hallucinations as the patient tries to sleep. Finding of cataplexy, sudden loss of muscle tone upon experiencing a strong emotional stimuli, is not as common but pathognomonic for narcolepsy [11]. Based on his clinical history of EDS, cataplexy coupled with his MSLT led to a diagnosis of narcolepsy despite lacking 2 SOREMs.

We presented a 42-year-old male who presented to our care in April 2018 for evaluation of sleep attack, snoring and hypnopompic/hypnagogic hallucinations dating as far back as adolescence. The patient described his quality of life being extremely poor especially because he worked as a taxicab driver which required utmost focus. Although the patient was able to work, his symptoms became a heavy burden exacerbated by not being able to keep his job. In addition to being undiagnosed, lack of work further challenged his quality of life. One of the most fascinating findings was when the patient referred to dozing off frequently while waiting for the traffic light to turn green. In addition to common narcolepsy findings, the patient shared his experience of lethargy as blood glucose level decreased.

Management of narcolepsy revolves around combination of pharmacologic and non-pharmacologic agents tailored to the patient based on severity of their condition. In addition to medication, the patient must demonstrate the ability to stay awake during a maintenance of wakefulness test (MWT). This test is used to quantify the patient's ability to remain awake during four to five sessions of 40 minutes in a quiet, relaxing and stimulation-free environment [12]. To pass this test, the patient must stay awake for a minimum of 40 minutes during each trial which was achieved by our patient. Furthermore, during the last visit, the patient expressed complete satisfaction with the management provided for narcolepsy and sleep apnea, denying any EDS or cataplexy attacks since the treatment began. Moreover, he was cleared to drive again with serial visits to confirm his continuous compliance with CPAP and medications.

There are many reasons for presenting this case, the most important is to bring awareness about this condition. Clinical data suggests that there is a significant delay in diagnosis with the mean age being 10 to 15 years from onset of symptoms and this is especially true for this patient [13]. A recent study suggests that annual direct medical costs associated with narcolepsy and matched cohort of patients without narcolepsy is $11,702 and $5,261 (p < 0.0001), respectively [14].

## Conclusions

Narcolepsy is a challenging condition to diagnose and treat and the impact on a patient is often difficult to quantify. A patient may have reduced day to day functionality and may be subjected to multiple evaluations by specialist as he or she attempts to put a name to their symptoms which may dramatically affect quality of life at work or at home. Our patient struggled with his condition since childhood and there is noway of determining how much different he would have been socially, mentally, and physically if a diagnosis was rendered at a younger age. Fortunately, an official diagnosis of narcolepsy and obstructive sleep apnea was made which led to aggressive treatment and a good outcome for the patient. He likely has a better probability of enjoying a normal life or at least a more normal routine as he stays focused on life's goals.
